# Supplementary material for: Diurnal Changes in Water Soluble Carbohydrate Components in Leaves and Sucrose Associated TaSUT1 Gene Expression during Grain Development in Wheat
Source: Int J Mol Sci. 2020 Nov 5;21(21):8276. doi: 10.3390/ijms21218276 (PMC7663803; doi:10.3390/ijms21218276)
Supplement: Supplementary file 1 [file ijms-21-08276-s001.pdf]

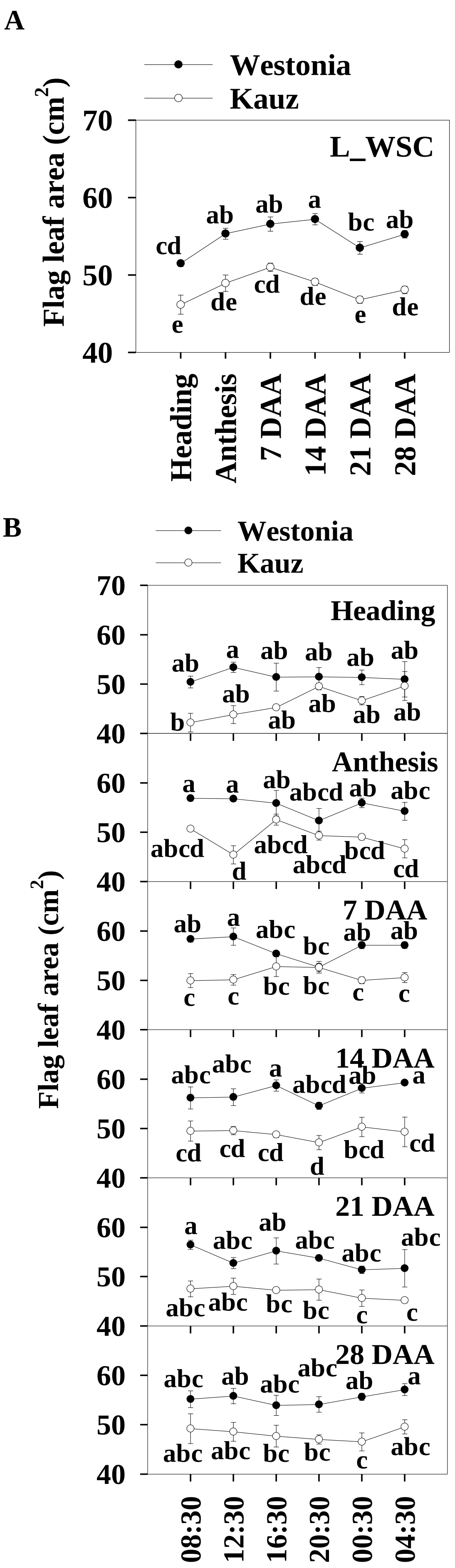

Figure S1. Flag leaf area and its diurnal data across grain developmental stages in Westonia and Kauz. (**A**) Flag leaf area across grain developmental stages; (**B**) Flag leaf area diurnal data during grain development. The vertical bars represent SE. Values with the same letter are statistically not different at $P = 0.05$.

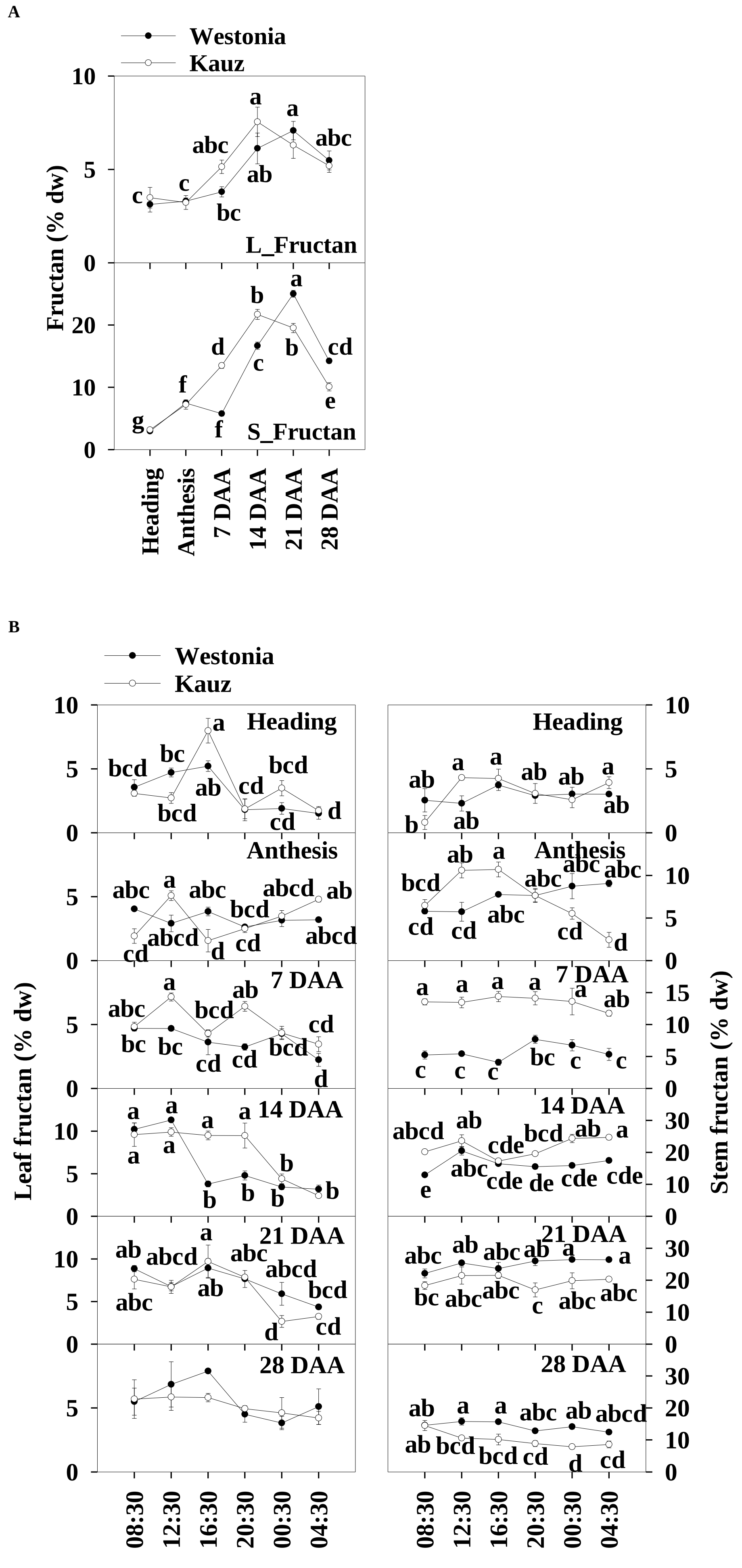

Figure S2. Fructan concentrations and its diurnal patterns in the flag leaves and main stems across grain developmental stages in Westonia and Kauz. (**A**) Fructan concentrations in the flag leaves (L_Fructan) and main stems (S_Fructan) across grain developmental stages in Westonia and Kauz; (**B**) Fructan diurnal analysis in the flag leaves (left) and main stems (right) of Westonia and Kauz during grain development. The vertical bars represent SE. Values with the same letter are statistically not different at $P = 0.05$.

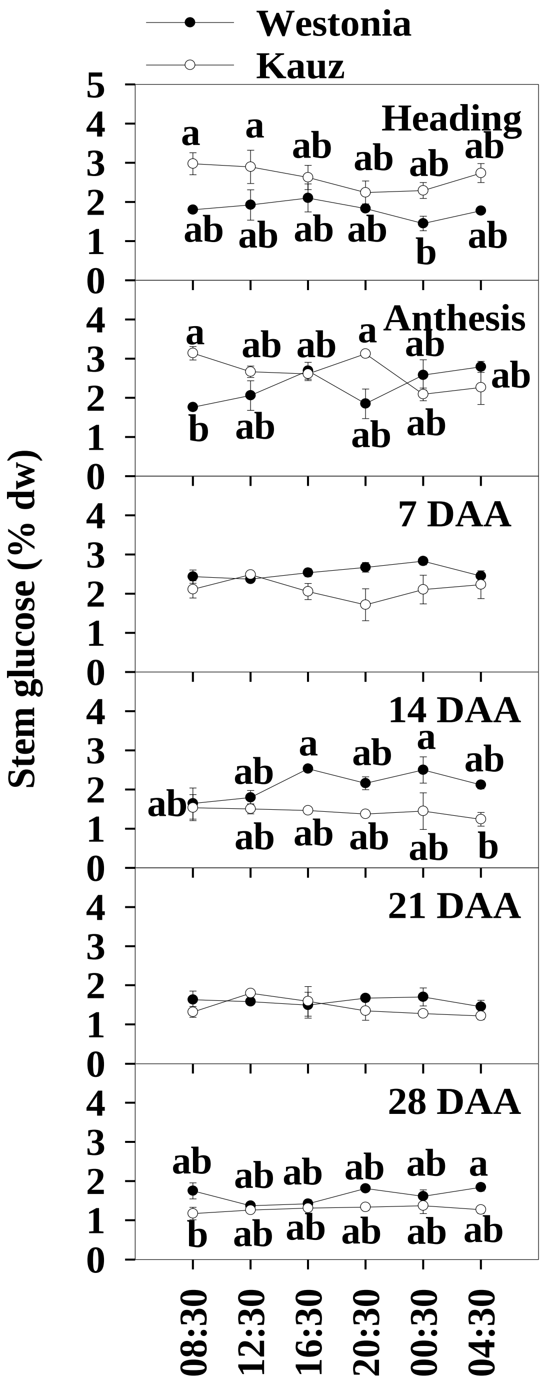

Figure S3. Glucose diurnal analysis in the main stems of Westonia and Kauz during grain development. The vertical bars represent SE. Values with the same letter are statistically not different at *P* = 0.05.

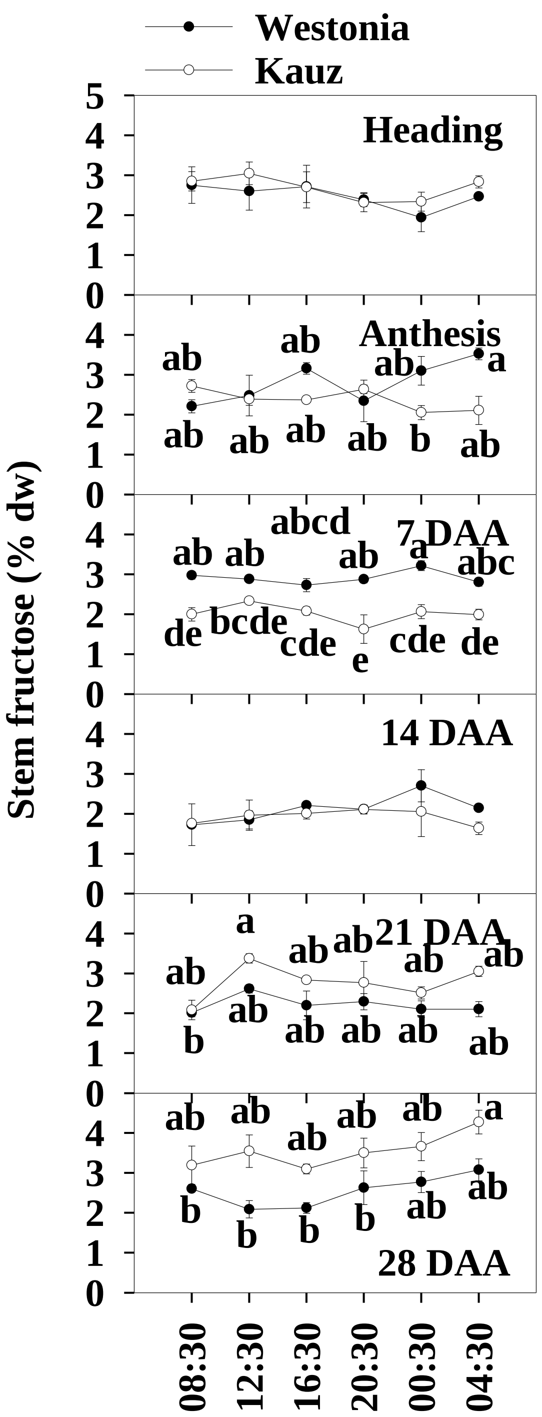

Figure S4. Fructose diurnal analysis in the main stems of Westonia and Kauz during grain development. The vertical bars represent SE. Values with the same letter are statistically not different at $P = 0.05$.

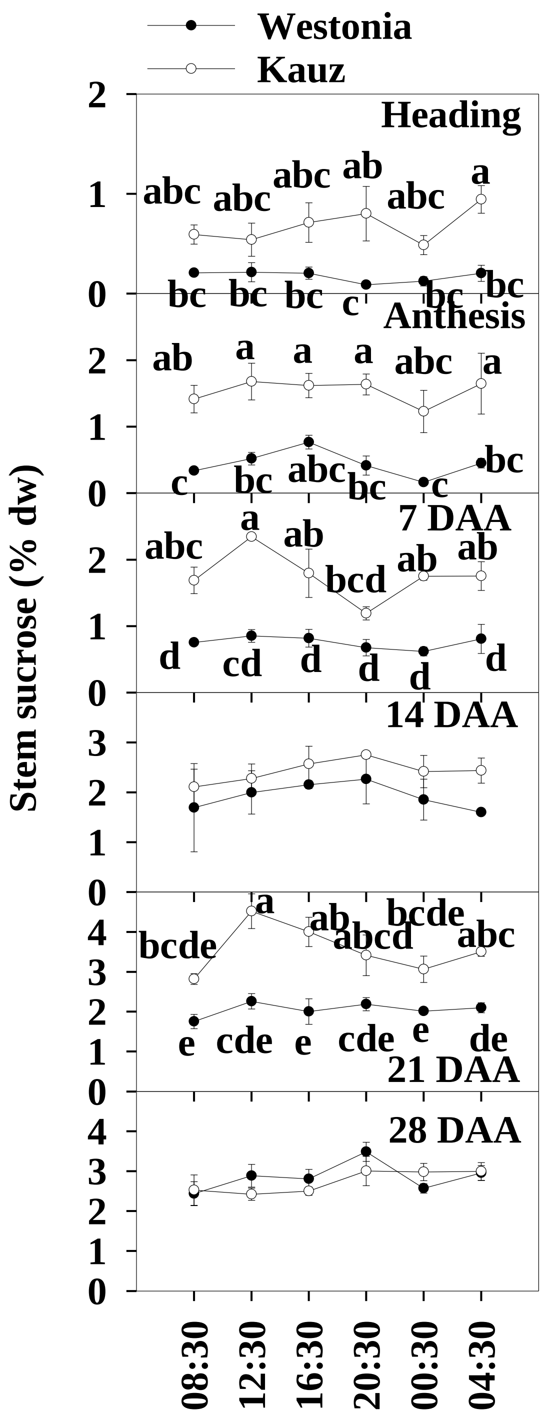

Figure S5. Sucrose diurnal analysis in the main stems of Westonia and Kauz during grain development. The vertical bars represent SE. Values with the same letter are statistically not different at $P = 0.05$.

A

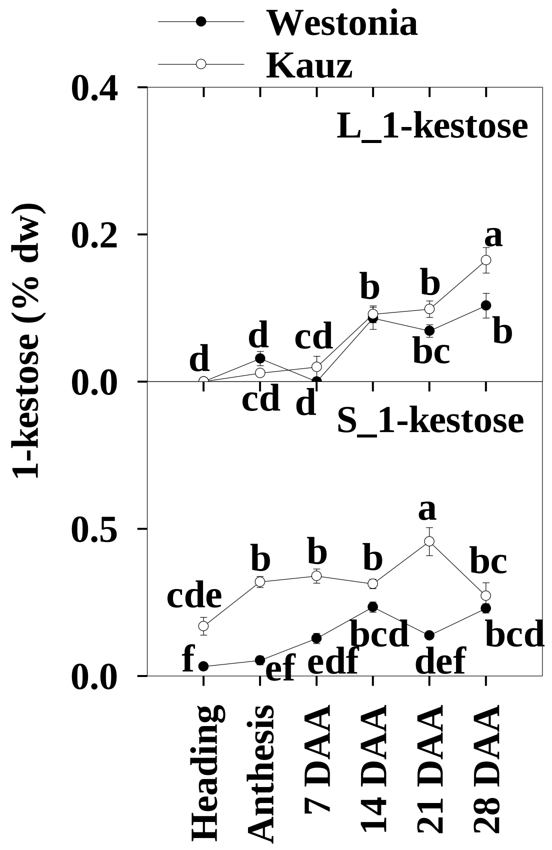

B

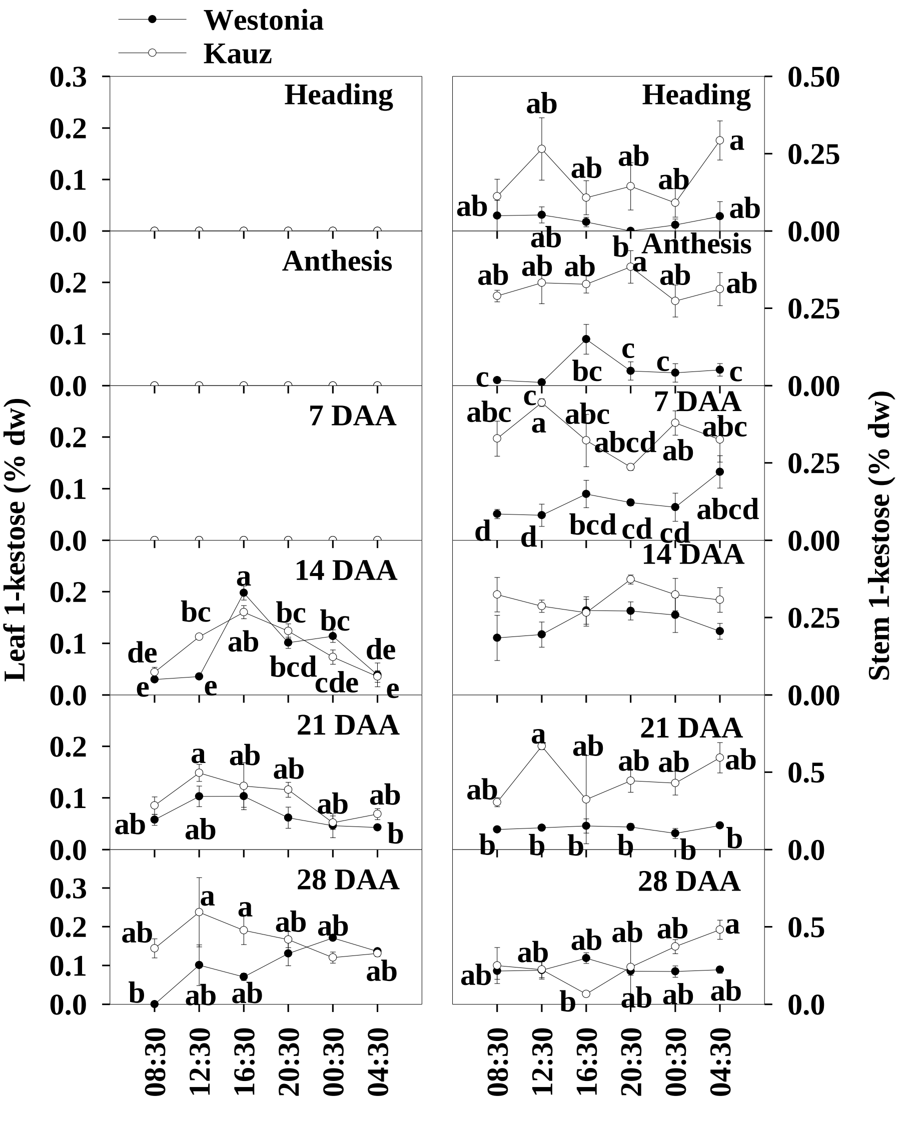

Figure S6. 1-kestose concentrations and its diurnal patterns in the flag leaves and main stems across grain developmental stages in Westonia and Kauz. (**A**) 1-kestose concentrations in the flag leaves (L_1-kestose) and main stems (S_1-kestose) across grain developmental stages; (**B**) 1- kestose diurnal analysis in the flag leaves (left) and main stems (right) of Westonia and Kauz during grain development. The vertical bars represent SE. Values with the same letter are statistically not different at P = 0.05.

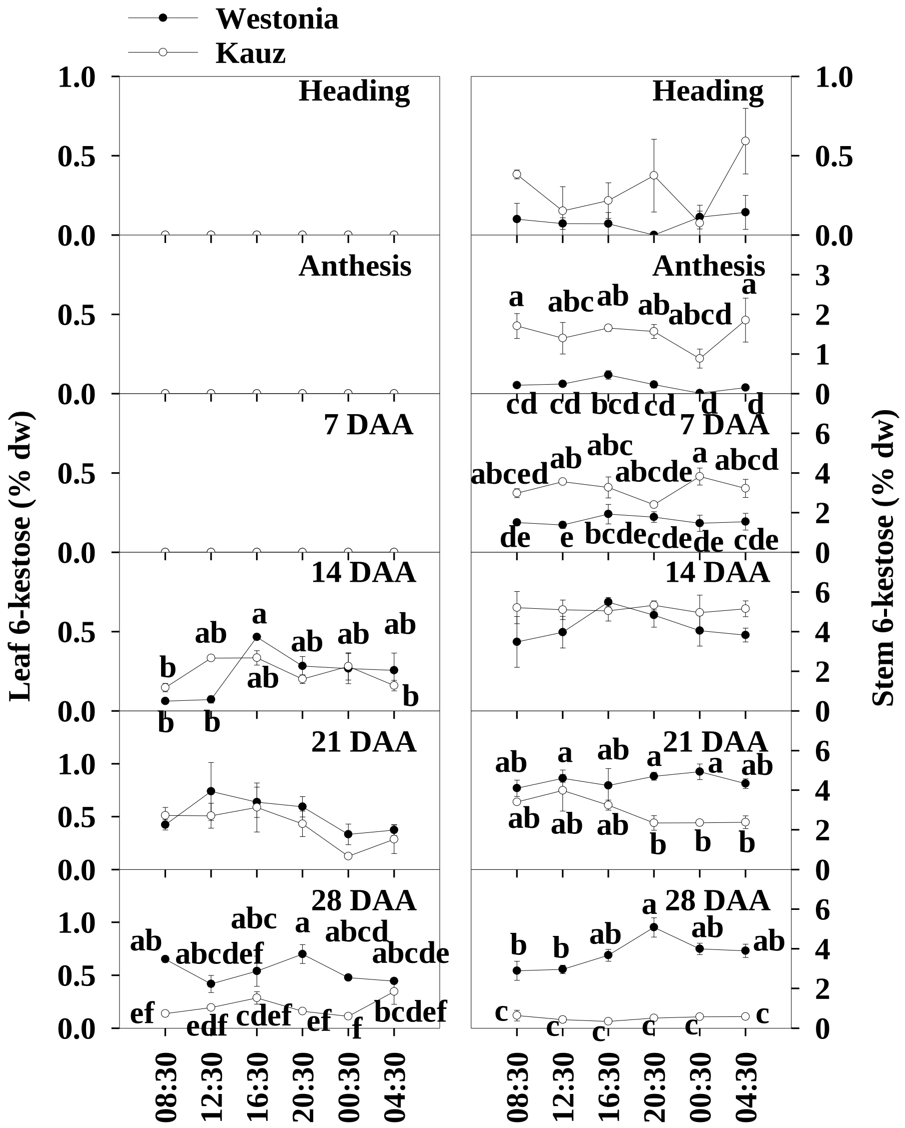

Figure S7. 6-kestose diurnal analysis in the flag leaves (left) and main stems (right) of Westonia and Kauz during grain development. The vertical bars represent SE. Values with the same letter are statistically not different at $P = 0.05$.

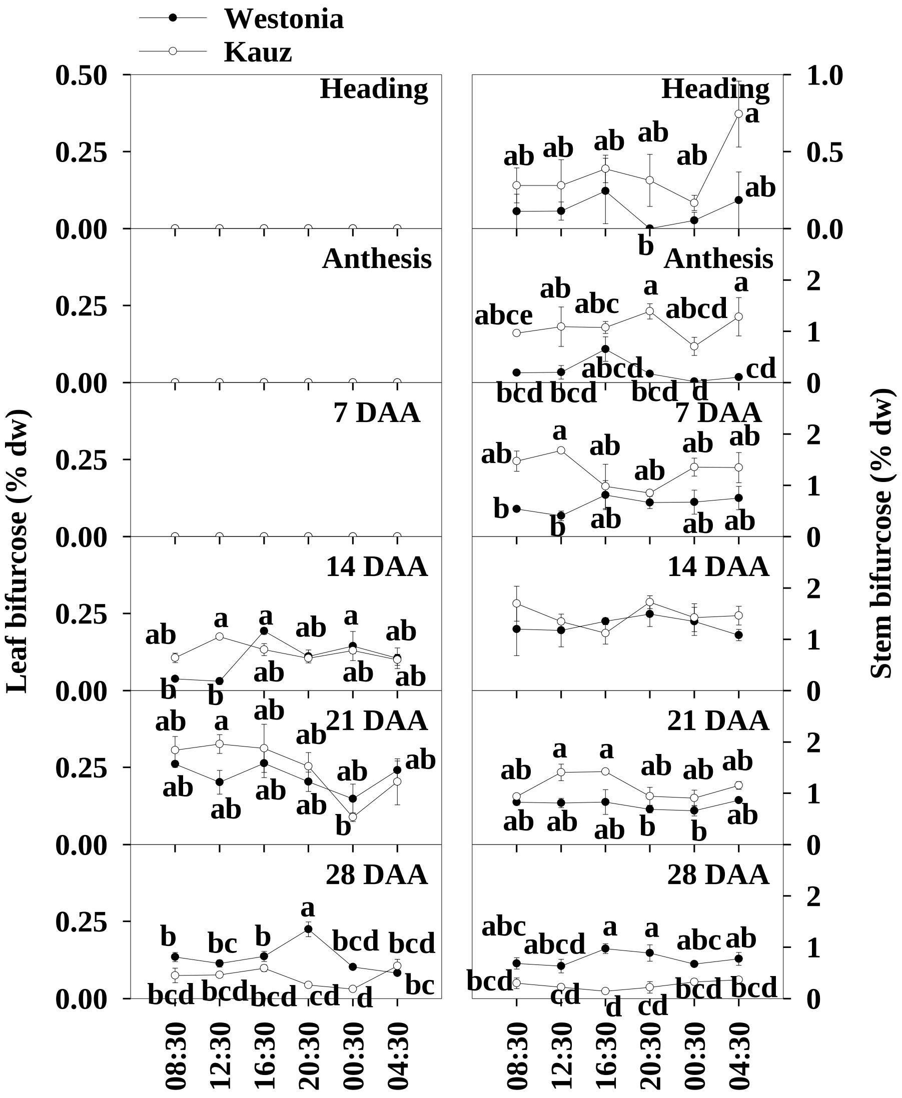

Figure S8. Bifurcose diurnal analysis in the flag leaves (left) and main stems (right) of Westonia and Kauz during grain development. The vertical bars represent SE. Values with the same letter are statistically not different at $P = 0.05$.

A

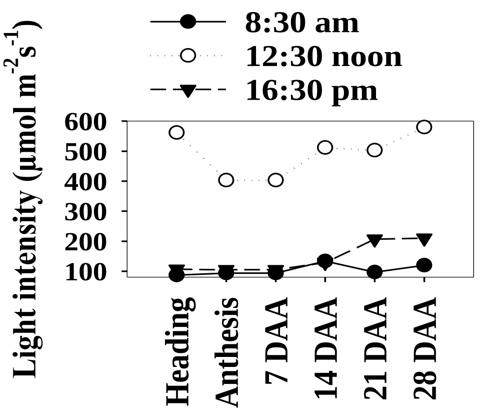

B

Day time	Heading	Anthesis	7 DAA	14 DAA	21 DAA	28 DAA
Sunrise	06:46	06:37	06:33	06:18	06:11	06:00
Sunset	17:53	17:58	17:58	18:07	18:10	18:20
Day length (h)	11:07	11:21	11:25	11:49	11:59	12:20

C

Sampling time	Heading	Anthesis	7 DAA	14 DAA	21 DAA	28 DAA	35 DAA
08:30	19.16	20.23	18.89	19.59	22.28	21.03	21.91
12:30	22.09	21.79	21.88	21.82	22.37	22.25	21.76
16:30	20.42	17.30	20.96	21.58	21.85	21.73	21.76
20:30	13.85	12.72	15.20	15.01	15.59	15.81	18.16
00:30	12.20	11.68	13.97	13.85	13.03	13.30	16.97
04:30	11.47	10.80	12.42	13.30	11.44	11.56	14.89

Figure S9. Light intensity at 8:30 am, 12:30 noon and 16:30 pm (**A**), the sunrise, sunset and day length (**B**) and (**C**) the temperature (℃) during sampling time across grain developmental stages.

A

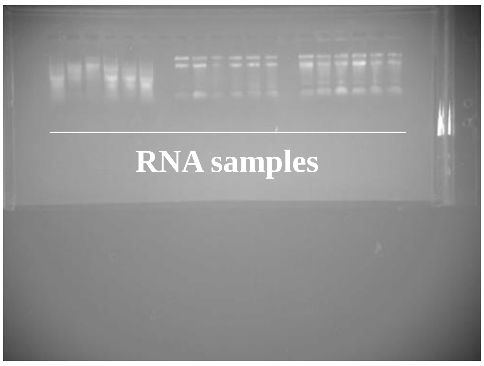

B

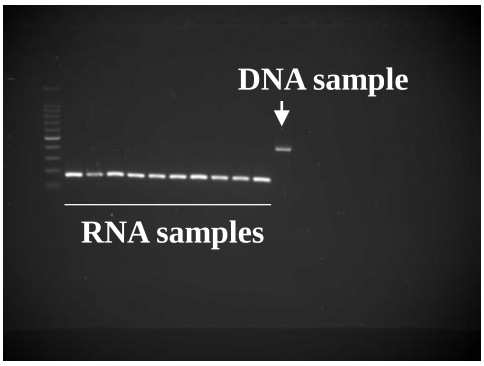

Figure S10. Extracted RNA running on 1.5% agarose gel electrophoresis (**A**) and DNA was removed from total RNA extracts by treatment with RNase free DNase I in a *GAPDH* amplification (**B**).
